# The Common Marmoset as a Novel Non-human Primate Model for Inner Ear Research

**DOI:** 10.31662/jmaj.2025-0142

**Published:** 2025-05-30

**Authors:** Makoto Hosoya

**Affiliations:** 1 Department of Otorhinolaryngology-Head and Neck Surgery, Keio University School of Medicine, Tokyo, Japan

**Keywords:** common marmoset, hearing loss, cochlea, primate model

## Abstract

Recent advances in molecular biology have led to significant progress in the fields of otology and audiology. Rodents, particularly genetically modified mice, have traditionally served as the primary model for inner ear research. However, growing evidence highlights inter-species differences in hearing research. Simultaneously, the use of human inner ear specimens has become increasingly restricted due to difficulties in specimen collection and ethical concerns. Similarly, the use of human fetuses to study inner ear development is challenging due to ethical issues. Therefore, the embryology of the mammalian inner ear cochlea has been studied using rodent models. These challenges underscore the need for a new research platform that better approximates the human inner ear. The common marmoset (*Callithrix jacchus*), a New World monkey native to South America, has emerged as a promising alternative. Initially studied in adult models, this primate is now being applied to developmental inner ear research. Its use is expected to yield novel insights. Offering a viewpoint distinct from conventional rodent-based studies. In this study, we outline the advantages of the common marmoset in hearing research and discuss its potential as a primate model animal for future inner ear studies.

## Introduction

Understanding the pathophysiological mechanisms of diseases is essential for advancing medicine, driven by the accumulation of medical and scientific knowledge. In otology and audiology, molecular biological techniques have led to significant discoveries. Like other fields, inner ear research relies heavily on cell lines and animal models ^(1), (2)^. Rodents, birds, fish, and other animals have been widely used as model organisms ^(3), (4)^, with transgenic mice serving as a particularly versatile and informative tool for inner ear research ^(5)^. However, there are many limitations to these models, creating a need for research from an alternative viewpoint different from conventional methods.

Unlike research on abdominal organs, inner ear studies face unique challenges. Biopsies of pathological inner ear cells are rare, as they lead to profound and irreversible hearing loss. Therefore, opportunities to observe human inner ear cells are limited. Attempts have been made to utilize rare human cadaveric temporal bone specimens; however, these are mainly used for anatomical and histological studies and are seldom used for molecular biological purposes in Japan and internationally. Moreover, opportunities for basic research using cadaveric human temporal bone specimens are limited, especially in Japan. These limitations highlight the difficulty of using human cells and tissues in inner ear research, emphasizing the need for an alternative research platform ^(6)^.

As mentioned above, rodent models remain one of the most powerful tools for investigating ear pathophysiology ^(7)^. However, inter-species differences between rodents and primates have been reported ^(8)^, and several issues remain unsolved in rodent-based studies. Against this background, we have focused on the common marmoset, a small primate, as a model and have researched to establish it as a new platform for inner ear studies. With recent advances in molecular biological approaches, the common marmoset has gained attention as a promising model for inner ear research. In this paper, we summarized current findings from common marmoset-based inner ear research and outlined future perspectives.

## The Common Marmoset as a Small Primate Model Animal

The common marmoset (*Callithrix jacchus*) is a New World monkey species native to South America ^(9)^. An adult marmoset measures approximately 20-25 cm and weighs 250-500 g. In the wild, it is found primarily found in northeastern Brazil ^(9)^.

**Figure 1.**
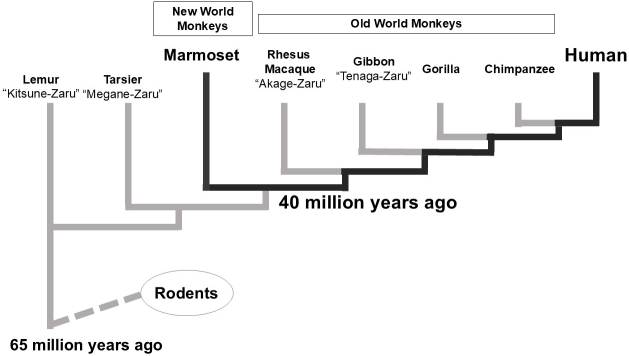
Primate phylogenetic tree. The common marmoset of New World monkeys is more closely related to humans than to rodents, followed by Old World monkeys.

Evolutionarily, the common marmoset is more closely related to humans than rodents, although it is less closely related to Old-World monkeys (Figure 1). While larger primates, such as gorillas, orangutans, and chimpanzees―more closely related to humans―are endangered, the common marmoset has a stable wild population. Old-world monkeys are not at risk of extinction; however, their larger size makes them less suitable as laboratory animals. In contrast, the common marmoset is suitable for use as a laboratory animal.

Compared with rodents, the common marmoset is genetically closely related to humans while remaining smaller and easier to handle among primates. It also exhibits high reproductive efficiency and is well-suited for developmental engineering. Its entire genome has been sequenced, facilitating molecular biological research ^(10)^. Notably, it was the first primate for which genetically modified individuals were successfully produced, with reports dating back to 2009 ^(11)^. Another significant advantage is its accessibility in Japan, where breeding colonies have been established for laboratory use.

Traditionally, common marmosets have been primarily used as laboratory animals for research on the central nervous system and behavioral analysis ^(12)^. While primates and rodents differ significantly in size, structure, and neurophysiological mechanisms of central nervous systems, the common marmoset has gained attention due to its homology with humans. This makes it particularly valuable in areas such as central nervous system and psychiatric disease research, where rodents are not readily available, thus minimizing the gap between human patients and animal models.

In otolaryngological research, the common marmoset offers several advantages. It exhibits human-associated traits, including complex vocalizations and verbal communication ^(13)^. Moreover, its hearing range overlaps with that of humans ^(14)^, and cochlear implantation has been successfully applied to this species ^(15), (16), (17)^, highlighting its potential as a hearing research model. With the advent of genetic modification techniques in common marmosets ^(11), (18), (19)^, this species is suitable for investigating the detailed pathogenic mechanisms of genetic hearing loss. Anatomical evaluations of the middle and inner ear have also been reported, particularly in studies involving drug administration ^(20), (21)^. These studies suggest the marmoset’s potential for evaluating new therapies in pre-clinical trials.

## Cochlea of the Common Marmoset

The cochlea is an essential organ for hearing. Sound waves are converted into neural signals by hair cells in the cochlea, located within the temporal bone. These electro-neuronal signals reach the brain via spiral ganglion neurons, where they are perceived as sound. This pathway constitutes the process of hearing.

The common marmoset cochlea, like that of humans, has 2.75 turns, divided into basal, middle, and apical regions ^(22)^. In contrast, rodents exhibit varying numbers of cochlear turns: mice have 1.75-2 turns, rats have 2.1 turns, and guinea pigs have 3.5 turns ^(23)^. The morphological similarity between the human and common marmoset cochlea facilitates the translation of findings from this primate to human studies.

**Figure 2.**
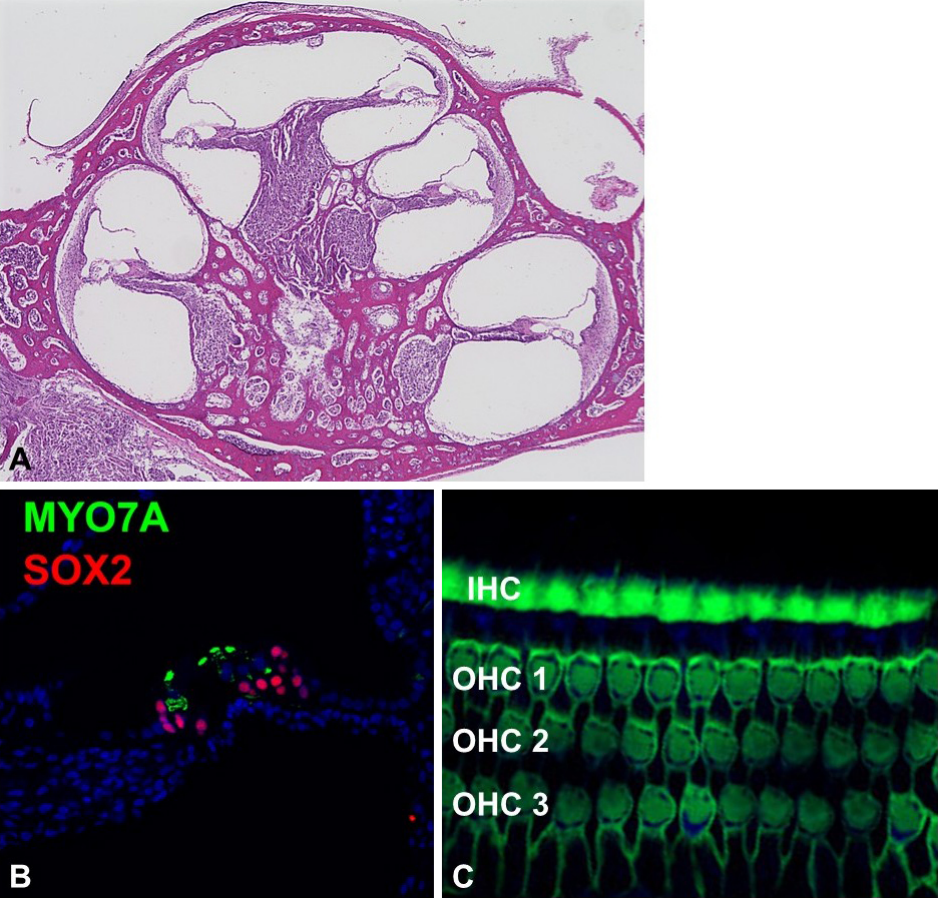
Histology of the newborn cochlea of the common marmoset. The newborn cochlea of the common marmoset has 2 and a half turns, the same as the adult cochlear (A). Immunohistochemical analysis can be applied to this primate cochlea. One row of inner hair cells and 3 rows of outer hair cells can be clearly observed (B and C). IHC: inner hair cells, MYO7A: hair cell markers; OHC: outer hair cells; SOX2: supporting cell markers.

Molecular biological techniques applicable to rodent models are also available for the common marmoset cochlea (Figure 2). With its genome and amino acid sequences fully determined, deoxyribonucleic acid, ribonucleic acid, and protein expression can be analyzed using standard methods such as polymerase chain reaction and Western blotting. In addition, immunohistochemical techniques for cochlea analysis have been established for temporal bone studies ^(24), (25)^. For histological analysis, the common marmoset’s smaller temporal bone offers the advantage of short decalcification times, which helps preserve antibody reactivity compared with larger primates, including humans.

## Genetic Hearing Loss Research Using Common Marmosets

Genetic hearing loss is a type of hearing loss ^(26)^ caused by variants in specific genes, leading to various forms of sensorineural hearing loss in either recessive or dominant patterns. Over 100 causative genes have been identified ^(26)^. Traditionally, research on genetic hearing loss has relied on genetically modified mouse models to replicate human hearing loss pathologies and analyze their underlying mechanisms ^(5), (27), (28)^. However, some causative genes associated with human hearing loss fail to reproduce the hearing loss phenotype in genetically modified mice, highlighting potential inter-species differences in the pathophysiological mechanisms of genetic hearing loss ^(29)^. Therefore, our initial studies focused on hearing loss genes expected to exhibit inter-species differences between rodents and primates ^(22), (30), (31), (32)^.

Using immunohistological techniques, we examined adult common marmoset cochlear tissue to identify expression patterns of typical marker genes in hair cells, supporting cells, and the stria vascularis ^(22)^. We specifically targeted causative genes that could not replicate human hearing loss phenotypes in mouse models ^(22)^, and compared their expression patterns between rodents and common marmosets ^(22), (30), (32)^. Our findings revealed that some causative genes, which fail to reproduce human hearing loss in rodents, exhibit different expression patterns in primates.

For example, mouse models struggle to replicate human hearing loss phenotypes caused by mutations in *CX31* ^(22)^, *DFNA5* ^(22)^, *WFS1* ^(31)^, and *EYA4* ^(32)^. In contrast, the gene expression patterns in the common marmoset cochlea differ significantly from those in mice. These studies revealed that, for at least some genes, cochlea gene expression patterns vary greatly between rodents and primates.

These inter-species differences may explain why conventional mouse models often fail to replicate human hearing loss phenotypes. This observation underscores the value of the common marmoset as a model for genetic hearing loss research. For instance, the expression pattern of the* EYA4* gene, which is associated with a relatively high frequency of hereditary hearing loss, was later examined in human temporal bones. The results showed that the gene expression pattern in humans resembles that in the common marmoset, unlike in rodents ^(33)^. This example highlights the similarity between common marmosets and humans and demonstrates the usefulness of this animal for preliminary studies before using scarce human temporal bone specimens.

The establishment of genetic modification technology in common marmosets ^(18)^ makes it theoretically possible to create genetically modified marmosets with genetic hearing loss. In addition, since the common marmoset has a lifespan of over 10 years, it is suitable for research on slowly progressive hearing loss models, which are challenging to investigate in mice. Further effective utilization in this research field is expected.

## Developmental Study of the Cochlea Using Common Marmosets

We extended our research on the common marmoset to the field of developmental studies ^(25)^. Developmental findings are an important scientific topic, providing foundational knowledge for regenerative and stem cell medicine. In many regenerative medicine approaches, the goal is to mimic the developmental organ formation processes of the target cells to regenerate lost cells. For example, hair cells in the inner ear are an important target for regenerative medicine. Several attempts have been made to induce the hair cells from surrounding supporting cells, which share common developmental progenitor cells with hair cells ^(34)^. Similarly, when inducing hair cells or spiral ganglion neurons from pluripotent stem cells, such as induced pluripotent stem (iPS) cells, the fundamental approach is to understand how these cells are formed during development and to replicate this process* in vitro* ^(35), (36)^.

To date, the developmental biology of the mammalian inner ear has been primarily studied in rodent models, especially mice ^(37)^. However, studies on human fetuses have highlighted embryological differences between rodents and humans ^(38), (39)^. Research using human fetuses is extremely limited due to ethical concerns, particularly because the major structures of the inner ear form relatively late in human embryonic development. For example, hair cell formation begins around 12 weeks of gestation, while spiral ganglion neuron maturation and stria vascularis formation occur even later, with full inner ear maturation estimated at around 30 weeks of gestation ^(40), (41), (42)^. These timelines coincide with ethical challenges, such as the timing of abortion, making it difficult to use human fetuses for developmental studies globally ^(42)^.

Due to these ethical constraints, analyses of inner ear development in human fetuses rely on historical specimens. However, these specimens are often unsuitable for modern molecular biological techniques due to limitations in fixation and storage methods. Therefore, studies using human fetal cochlear specimens are often limited to histological and morphological evaluations.

In contrast, recent advances in molecular biology have made inner ear regenerative medicine a reality. The integration of human embryonic stem/iPS cell research into the field has increased the demand for detailed knowledge of human inner ear development. For future clinical applications of regenerative medicine, it is essential to identify differences in inner ear development between rodents and primates and their significance. While human fetal specimens remain inaccessible, the medical and scientific importance of elucidating human inner ear development is increasing. This underscores the need for a primate model that can serve as an alternative platform for developmental research in otology.

To address this need, we investigated cochlear development in common marmoset fetuses from embryonic day 70 to birth (approximately embryonic day 150) ^(43)^. We aimed to identify similarities and differences between these primate and conventional rodent models ^(25), (44)^. Previous studies have demonstrated that the inner ear development of this animal closely resembles that of humans, suggesting it can serve as an alternative to human fetal studies ^(25)^.

## Development of the Sensory Epithelium in the Common Marmoset

**Figure 3.**
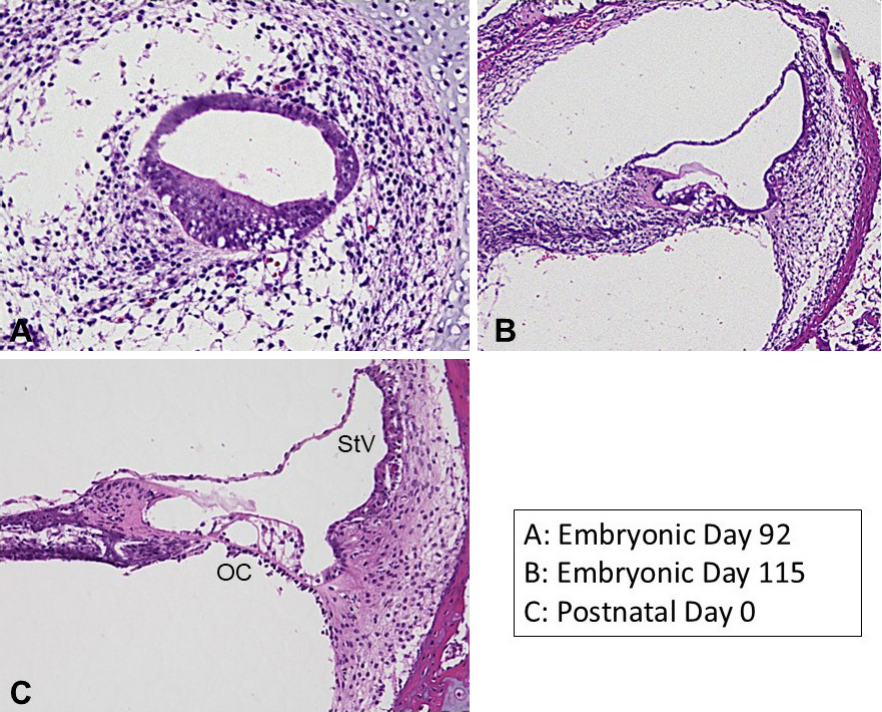
Developmental, morphological changes of the cochlea of the common marmoset. During the cochlear development, the cochlear duct changes its morphology. Previous studies on developmental investigation in primate cochlea have described their general time course. The common marmoset has become a novel developmental study model animal in this field. OC: organ of Corti, StV: stria vascularis.

The cochlea undergoes significant morphological changes during development (Figure 3). Previous studies have shown that in the 70-day-old fetus, the cochlea does not yet exhibit its characteristic coiled structure but instead appears as a hooked formation ^(44)^. As development progresses, the cochlea begins to coil, and by embryonic day 92, it forms a 2-and-a-half turn structure similar to that of the adult. Concurrently, the sensory epithelium develops, mirroring the process observed in other model animals.

Previous studies have revealed that gene expression patterns in cochlear hair cells are relatively conserved between rodents and primates. However, significant inter-species differences have been detected in supporting cells and spiral ganglion neurons ^(25)^. For example, when examining the expression patterns of *CDKN1B*, *GATA3*, *SOX2*, *SOX21*, *ISL1*, *AQP4*, *FGFR3*, and *CD44* genes―which are known as supporting cell markers in the cochlea―the spatiotemporal expression patterns of these markers were found to differ, at least partially, from those in rodents during development.

Inter-species differences between rodents and primates have also been reported in key signaling pathways for cochlear development ^(45)^. For example, Notch signaling is known to play a vital role in the development of the organ of Corti. Hair cells and supporting cells differentiate from common precursor cells in the cochlear sensory epithelium, and Notch signaling is essential for inducing this differentiation. This signaling pathway is also of particular interest from the perspective of hair cell regeneration ^(46)^.

We investigated the expression of Notch signaling during the differentiation of hair cells and supporting cells in the common marmoset ^(45)^. Notably, in rodents, *Jag1* expression precedes *Sox2* expression during the domain-determining phase in the sensory epithelium. In contrast, in the common marmoset, *SOX2* expression precedes *JAG1* expression. This gene expression pattern observed in the common marmoset was later confirmed to be similar in human fetuses, indicating a unique primate-specific gene expression pattern ^(47)^. Furthermore, the activation of *NOTCH1* in supporting cells and the expression of the *HES1* gene, downstream of *NOTCH1*, persist longer in the common marmoset compared to rodents ^(45)^. This observation aligns with the previously noted inter-species differences in gene expression patterns in supporting cells, indicating potential differences in the regulatory mechanisms of gene expression between hair cells and supporting cells in rodents and primates. These differences warrant further investigations in future studies.

Supporting cells and spiral ganglion neurons are considered critical targets for regenerative medicine. Therefore, the findings from the common marmoset are highly relevant for future human inner ear regeneration. Specifically, while the properties of cochlear hair cells are relatively conserved between rodents and common marmosets, significant inter-species differences may exist in the properties of supporting cells and spiral ganglion neurons ^(25), (48)^. Given that hair cell regeneration often relies on supporting cells, these inter-species differences could significantly impact inner ear regeneration capacity. This highlights the importance of considering such differences when translating data from rodent models to human clinical applications in the future.

## Development of the Spiral Ganglion Neurons in the Common Marmoset

Spiral ganglion neurons are essential in transmitting neuroelectric signals, generated by the mechanoelectrical conversion in hair cells, to the central nervous system. The development of spiral ganglion neurons in the common marmoset cochlea has been previously studied ^(25), (44), (48)^.

The development of spiral ganglion neurons occurs in parallel with the elongation and coiling of the cochlear duct ^(44), (48)^. At embryonic day 70, spiral ganglion neurons are not yet detectable. However, by embryonic day 77, they become observable. By day 87, glial cells appear around the spiral ganglion neurons. By day 96, the axons of the spiral ganglion neurons reach the sensory epithelium, where developing hair cells are located, although synapse formation with hair cells has not yet occurred. By embryonic day 101, shortly after hair cell formation, synapses between inner hair cells and the spiral ganglion neurons begin to form, starting from the basal turns. Between embryonic day 96 and day 115, numerous nerve endings are present around the hair cells. However, synaptic pruning occurs after embryonic day 115, resulting in a structure similar to the adult spiral ganglion neurons. Like the development of the sensory epithelium, the development of spiral ganglion neurons in the common marmoset takes significantly longer than in rodents. These findings provide valuable insights that could enhance our understanding of human hearing loss.

## Development of the Stria Vascularis and Lateral Wall Fibrocytes in the Common Marmoset

The stria vascularis, along with spiral ligament fibrocytes, plays an important role in establishing the electrolyte gradient within the cochlea. Histologically, the stria vascularis consists of 3 layers: marginal cells, intermediate cells, and basal cells. The development of the stria vascularis and adjacent spiral ligament fibrocytes has been studied in the common marmoset ^(49), (50)^.

In the common marmoset, the presumptive region of the stria vascularis within the cochlear duct can be identified by embryonic day 77 using the *PAX2* gene as a marker ^(44)^. However, the localization of the *BSND* gene (Barttin), a marker for marginal cells of the stria vascularis, is not detectable until around embryonic day 87. The differentiation of marginal cells begins in the cochlea’s basal turns and progresses toward the apical turn, with *BSND* gene expression in the apical turn observed around embryonic day 109 ^(49)^. At embryonic day 87, the stria vascularis is monolayered. As development progresses, *MLANA* (Melan-A)-positive intermediate cells and *IBA1*-positive macrophage-like cells migrate into the structure. The organization between intermediate and marginal cells becomes more complex, resembling the intricate structures seen in adults. By embryonic day 109, this complexity is evident. Around embryonic day 115, vascular structures penetrate the stria vascularis middle layer. At this stage, the *CLDN11* gene-positive tight junctions, characteristic of basal cells, are not yet observed but become evident at birth.

Currently, the common marmoset is the only primate model in which the embryonic development of the stria vascularis and spiral ganglion fibrocytes has been described from a molecular biological perspective ^(49), (50), (51)^. While studies on human fetuses are limited, comparisons indicate that the developmental timeline of the stria vascularis in the common marmoset closely mirrors that in humans. However, examining human fetuses, especially after 12 weeks of gestation, is ethically challenging. The slower developmental timing of the stria vascularis further complicates such studies in humans. Using a primate model with a developmental pace similar to humans is scientifically significant. The common marmoset not only serves as a substitute for human studies but also provides valuable preliminary insights for future human research.

## Changes in the Localization Distribution of Macrophages in the Common Marmoset Inner Ear

Macrophages play an important role in the immune response within the cochlea and have been associated with ototoxicity and tissue damage following acoustic trauma ^(52)^. Studies in rodents have documented changes in the localization of macrophages during cochlea development, highlighting their essential role in normal hearing ^(53), (54), (55)^. While similar changes have been reported in human cochlea development, knowledge in this area remains limited ^(56)^. The localization changes of macrophages during cochlear development in the common marmoset have also been previously investigated ^(57)^.

In the common marmoset cochlea, many cells express *IBA1*, a macrophage marker, surrounding the developing cochlear duct at embryonic day 70. At this stage, *IBA1*-positive cells are primarily located among *POU3F4* gene-positive mesenchymal cells (periotic mesenchyme), with some invading the epithelium. These *IBA1*-positive cells remain abundant around the peri-cochlear duct until approximately embryonic day 96, rapidly declining through embryonic day 109. By birth, *IBA1-*positive cells are sparsely observed in the lateral wall fibrocytes, stria vascularis, spiral ganglion neurons, and the organ of Corti. Within the stria vascularis, *IBA1*-positive cells are present around blood vessels.

The common marmoset is the only non-human primate in which the localization of macrophages during cochlear development has been studied. The localization of macrophages is generally similar to that observed in rodents and humans during cochlea development ^(57)^. In both rodents and primates, *IBA1*-positive cells are abundant in the cochlea during early development but decrease in number as development progresses. Macrophages are implicated not only in the immune response within the cochlea but also in inner ear disorders. The ability to visualize macrophages in the common marmoset and apply insights from developmental processes to studies of aging and immunity holds significant promise for future research.

## Slower Speed Is Beneficial in the Observation of the Development of the Cochlea

Studies using the common marmoset have revealed new findings not previously reported in rodent models. For example, the *SLC12A2* gene (encoding Na^+^-K^+^-Cl^-^ cotransporter [NKCC1]), which is known to be expressed in the stria vascularis and lateral wall fibrocytes, is also transiently expressed in the organ of Corti during inner ear development ^(25)^. Furthermore, the expression pattern of genes involved in synaptic vesicle exocytosis in hair cells exhibits diverse temporal changes during development ^(25), (48)^. In addition, the subcellular localization of connexin26 and connexin30, proteins associated with hereditary hearing loss, has been shown to undergo significant changes during development ^(58)^.

Inner ear studies in the common marmoset not only confirm known biological phenomena observed in humans and mice but also enable time-resolved analysis of transient gene expression patterns and time-dependent biological changes. We believe these novel findings are partly attributable to the slower pace of inner ear development in the common marmoset compared with rodents. The common marmoset takes approximately 3 times longer to develop its cochlea, similar to humans ^(25)^. This slower developmental speed makes the common marmoset a suitable model for observing spatiotemporally transient phenomena during inner ear development, allowing researchers to identify details that may have been overlooked in previous studies using faster-developing rodent models.

## Future Prospects

It has become evident that findings from rodent studies cannot always be extrapolated to primates, even in cochlear development, which has often been considered identical between rodents and primates. This highlights the animal model’s future usefulness and importance. In addition, several findings from common marmoset studies have been later confirmed in human observations, underscoring its value as a screening and preliminary experimental model before studies using limited human samples ^(33), (47)^.

As summarized above, the common marmoset is currently the most advanced primate model for molecular biological analysis of inner ear development ^(6), (25)^. The development of various cochlear tissues has been extensively studied in this animal, including neurogenesis ^(48), (59)^, the development of the stria vascularis ^(49)^, lateral wall fibrocytes ^(50)^, and macrophage migration ^(57)^. Marker genes for each cell group have also been identified. These foundational studies in embryology are paving the way for further studies and applications in inner ear and hearing research, offering perspectives different from those of rodent models, which are frequently used in inner ear research.

**Figure 4.**
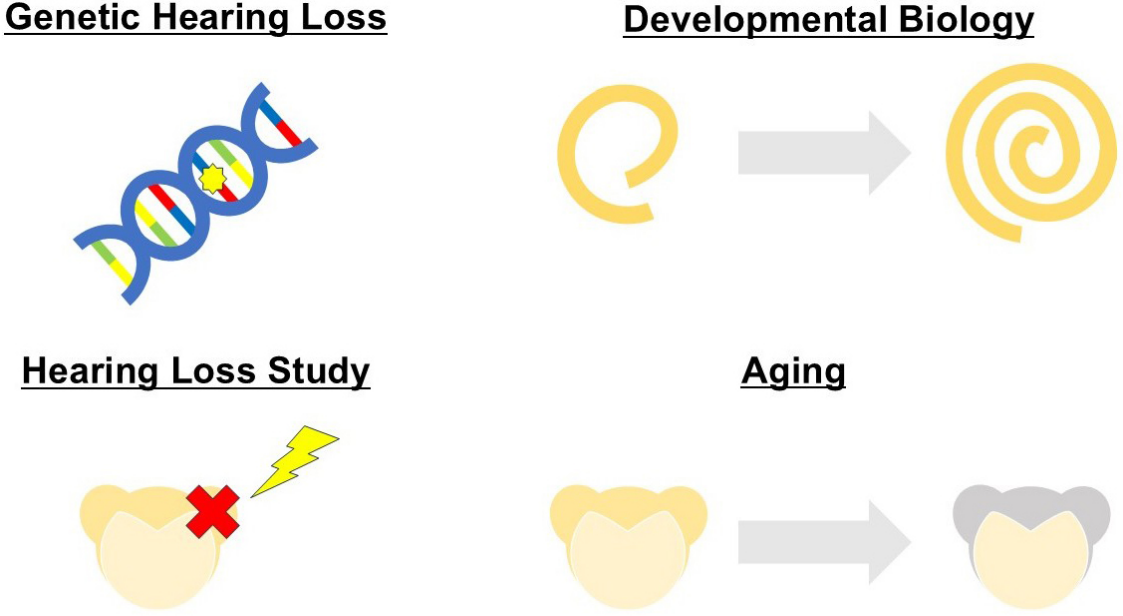
Usefulness of the common marmoset in hearing research. The common marmoset is a promising new animal model in the fields of otology and audiology. Common marmosets may be useful in studies of hearing loss and aging, as well as in studies of genetic hearing loss and developmental biology.

Furthermore, the common marmoset is expected to contribute to research on age-related hearing loss, acoustic trauma, drug-induced hearing loss, and other disorders, providing insights different from those obtained in rodents (Figure 4). Notably, this animal has a significantly longer lifespan than typical rodents and is known to experience age-related hearing loss ^(60)^. As described in this paper, the expression patterns of marker genes in cochlear cell populations during development and the inter-species differences have already been clarified. We believe these findings will establish a unique foundation for future research on age-related hearing loss, distinct from rodent-based research.

## Conclusion

This paper highlights the utility of the common marmoset as a novel model animal in otology and summarizes findings from studies on inner ear development using this primate. The knowledge gained from research on the common marmoset as a novel primate model for hearing loss and otology will be invaluable for investigating the pathophysiology of hearing disorders and developing novel therapies. The common marmoset represents a powerful tool in this field, and further studies are eagerly anticipated.

## Article Information

This article is based on the study, which received the Medical Research Encouragement Prize of the Japan Medical Association in 2024.

### Conflicts of Interest

None

### Sources of Funding

MH was supported by a grant from the Japanese government, MEXT KAKENHI (Grant-in-Aid for Scientific Research (B) 20H03836, Grant-in-Aid for Challenging Research (Exploratory) 21K19581, Grant-in-Aid for Scientific Research (C) 24K12727), the Keio Medical Association, and Keio University Medical Science Fund, Keio Gijuku Academic Development Funds, and Grant for Research Encouragement Award of the Japanese Society of Otorhinolaryngology-Head and Neck Surgery.

### Acknowledgement

The author thanks all colleagues for their constructive discussion and technical support.

### Approval by Institutional Review Board (IRB)

Animal experiments were approved by the Animal Experiment Committee of Keio University (approval numbers: 11006, 08020) and performed in accordance with the ARRIVE guidelines and the guidelines of the National Institutes of Health and the Ministry of Education, Culture, Sports, Science, and Technology of Japan.
